# Preliminary Exploration of Main Elements for Systematic Classification Development: Case Study of Patient Safety Incidents

**DOI:** 10.2196/35474

**Published:** 2022-03-29

**Authors:** Riikka Vuokko, Anne Vakkuri, Sari Palojoki

**Affiliations:** 1 Department of Steering of Health Care and Social Welfare Ministry of Social Affairs and Health Helsinki Finland; 2 Department of Anesthesiology, Intensive Care and Pain Medicine, Peijas Hospital Helsinki University Hospital Vantaa Finland

**Keywords:** classification, qualitative research, methodology, patient safety, validation

## Abstract

**Background:**

Currently, there is no holistic theoretical approach available for guiding classification development. On the basis of our recent classification development research in the area of patient safety in health information technology, this focus area would benefit from a more systematic approach. Although some valuable theoretical and methodological approaches have been presented, classification development literature typically is limited to methodological development in a specific domain or is practically oriented.

**Objective:**

The main purposes of this study are to fill the methodological gap in classification development research by exploring possible elements of systematic development based on previous literature and to promote sustainable and well-grounded classification outcomes by identifying a set of recommended elements. Specifically, the aim is to answer the following question: what are the main elements for systematic classification development based on research evidence and our use case?

**Methods:**

This study applied a qualitative research approach. On the basis of previous literature, preliminary elements for classification development were specified, as follows: defining a concept model, documenting the development process, incorporating multidisciplinary expertise, validating results, and maintaining the classification. The elements were compiled as guiding principles for the research process and tested in the case of patient safety incidents (n=501).

**Results:**

The results illustrate classification development based on the chosen elements, with 4 examples of technology-induced errors. Examples from the use case regard usability, system downtime, clinical workflow, and medication section problems. The study results confirm and thus suggest that a more comprehensive and theory-based systematic approach promotes well-grounded classification work by enhancing transparency and possibilities for assessing the development process.

**Conclusions:**

We recommend further testing the preliminary main elements presented in this study. The research presented herein could serve as a basis for future work. Our recently developed classification and the use case presented here serve as examples. Data retrieved from, for example, other type of electronic health records and use contexts could refine and validate the suggested methodological approach.

## Introduction

### Background

Classifications are applied for various purposes in clinical contexts, including patient documentation and more specific domains, such as the patient safety incident reporting of technology-induced errors. However, the availability of methodological frameworks and research evidence is limited for developing and maintaining clinical classifications [1-3]. Typically, classification development has practical goals in a specific clinical setting or documentation context. Accordingly, the results of practice-driven classification work are not necessarily transferable to other organizations, clinical domains, or purposes [4,5].

According to literature, severe challenges have arisen from the emergence of safety issues involving newly innovated and existing implemented health information technologies (ITs) [6-8]. Regarding patient safety in health IT, previous classification development-related research on technology-induced errors is available [3,9,10]. Magrabi et al [3] identified categories for populating the classification of IT problems with the aim of providing a clinical method for classifying computer-related patient safety incidents. This classification was then tested with patient safety incident data in a setting with 100% coverage of electronic health records (EHRs). The results indicated a need for classification development from the perspective of a sociotechnical approach [1]. Moreover, a recent study summarized that organizations today do not have rigorous, real-time approaches for routinely assessing the safety of EHRs or for identifying safety hazards [11]. This constitutes the underlying rationale for why we have recently developed and validated a classification of technology-induced error types from a social-technical perspective [12]. A guiding assumption in our research was that despite previous research introducing relevant themes in this area, further research is needed to capture the fast-developing clinical working environment. Health IT and EHRs advance in ways that require the identification and classification of new types of phenomena. Classifications evolve as new evidence arises [13].

On the basis of our experience when researching classification development, this focus area benefits from a more systematic approach. Although valuable theoretical and methodological approaches have been presented, some of the literature concentrates on a specific part of the classification development, such as validation within a specific domain [14] or maintenance and implementation of international classifications [15]. No holistic approach is provided to guide classification development. In addition, although some valuable research on patient safety incidents and respective classifications has been conducted, there is relatively scarce documentation of detailed classification development that could guide future development. It is suggested [16] that publishing methodological and theoretical approaches applied in classification development cases increases the validity and quality of development outcomes. This contributes in building a systematic approach to classification development. Currently, for example, some practical guidelines and web-based resources are available regarding large-scale international examples, such as the World Health Organization’s International Classification of Diseases and Systematized Nomenclature of Medicine–Clinical Terms [17,18]. However, the gap between practical guidelines and theory-based systematic classification development presents a challenge when the aim is to develop most sustainable, well-grounded classifications, which can also be assessed in a transparent way against a set of common development principles [16,19].

### Objectives

To benefit future classification work, in this paper, we present preliminary results on methodological considerations based on previous research evidence and our case research on classifying EHR-related patient safety incidents [12]. Our preliminary results are intended to inform future research on classification development research protocols with different use cases. We characterize typical elements of classification development and suggest a methodological approach [20] to achieve more systematic classification development after testing it with other use cases. Therefore, our main research question is as follows: what are the main elements for systematic classification development based on research evidence and our use case?

## Methods

### Evidence From Previous Research Informing Our Study Design

Sociotechnical theory has been a basis for the development of health IT-related models [21]. Pioneering theorists of the sociotechnical approach emphasized that technical and social systems should be optimized simultaneously and that organizations should comprise a relationship between nonhuman and human systems [22,23]. More specifically, the theoretical background for developing our classification was the Health IT Safety measurement framework proposed by Sittig and Singh [9,24]. The starting point of the Health IT Safety framework is that safety incidents must be understood within the full context of the *sociotechnical work system*. This refers to the interacting technical (eg, hardware and software) and nontechnical (eg, clinical workflow, people, and the physical environment) variables that affect health IT-related patient safety. The framework responds to the fundamental conceptual and methodological gaps related to both defining and measuring health IT-related patient safety. The aim is to provide a conceptual foundation for health IT-related patient safety measurement, monitoring, and improvement [10]. The following three domains were created to describe the range of risks and opportunities of health IT to influence patient safety: Safe Health IT, Safe Use of Health IT, and Using Health IT to Improve Safety. The latter includes using technology to identify and monitor patient safety incidents, risks, and hazards and to intervene before harm occurs [24].

EHR-related classifications for the purpose of incident reporting have been developed for specific clinical settings and problem areas. For example, the French Nuclear Safety Authority scale for classifying incidents was applied in oncology to inform the design and potential utility of an incident reporting system. All incidents during the research period were reviewed and graded according to potential severity by the consensus of a committee, including physicians and physicists [25]. However, the aim of the research was not to provide a detailed description of the development process of IT-related error-type categories from a methodological classification development perspective. Another example of classification-related development work in a specific IT-related error area is the development of a usability-related error ontology by Elkin et al [26]. Here, initial semantics for usability error types were derived from a literature review and an expert opinion. Then, a participatory design method was used to obtain input and feedback from multi-professional stakeholders. According to Elkin et al [26], with use and experience, the ontology would grow and evolve toward more standard and interoperable reporting. Intrinsically, ontologies have become important resources, because they can be applied to integrated EHR infrastructures to improve possibilities of data acquisition and storage, standardization, interoperation, data analysis for clinical research, and routine clinical documentation [27,28].

The classification developed by Magrabi et al [3] is a rare example of EHR-related classification development research that is not restricted to a specific EHR problem area. It was developed by using safety incidents and was further refined by analyzing data from incident reports submitted to a regulatory database. The methodology of classification development was based on the free-text descriptions of a quarter of the incidents retrieved (n=123), which were used to identify *natural categories* for the classification. A simple classification of the reported problems related to computer use was developed. The incidents were classified using the classification. An interrater reliability analysis was performed using the κ statistic to determine consistency among researchers. The classification developed by Magrabi et al [3] underwent testing in the United Kingdom after the preliminary development phases. In subsequent research, a limitation of the classification was documented: it was not possible to demonstrate the clinical relevance of all incidents by using this classification [29]. However, the study focused on case data–driven development of the classification, not on describing the underlying methodological aspects of classification theories.

In a recent study [12], a classification for patient safety incident reporting associated with the use of a mature EHR was developed, which was validated using a data set of 501 patient safety incidents. Here, a mature EHR is defined according to the Electronic Medical Record Adoption Model, which was developed by Healthcare Information and Management Systems Society Analytics. This universally recognized maturity model is an 8-stage model that reflects hospitals’ electronic medical record capabilities, ranging from a completely paper-based environment (stage 0) to a highly advanced paperless and digital patient record environment (stage 7). Regarding these data, the maturity level of the EHR system is 6 to 7 [12]. The classification development was based on research into commonly recognized error types. Further, a multi-professional research team used iterative tests on consensus building to develop a classification and preliminary descriptions of the classes. The final classification was validated using incident report data to evaluate its characteristics and applicability for purposes of patient safety incident reporting. The development focused on applying the theoretical aspects of classification development, for example, by defining concepts and the exclusiveness of categories and forming descriptions for all categories with the quality and usability of the resulting classification as a guiding principle. This classification development and validation research was used as a use case in this research to strengthen the fragmented methodological support for this type of research.

### Methodological Background and Study Design

This study applied a qualitative research approach that is applicable for identifying, characterizing, and interpreting a phenomenon. Qualitative methodology relies on data and their interpretation according to respective conceptualization [20,30]. To increase the reliability of qualitative research, applying a varied methodology is suggested [20,30-32]. For our use case [12], this involved retrieving clinical patient safety incident reporting data and conceptualizing technology-induced errors based on previous research, selecting relevant reports, analyzing incident data to define categories and hierarchies for the emerging classification, and applying a multidisciplinary panel within our research team to reach agreement over ongoing classification development (Multimedia Appendix 1). We have already documented the study design for data analysis and validation [12], and in this paper, our study design concentrates on specifically capturing classification development characteristics (Textbox 1) and related observations from the use case of our classification development. By creating a holistic methodological approach for classification development, there is potential to expand use to different kind of contexts and use cases.

**Main elements of classification development based on previous research.**

1. A concept model: a concept model supports the data analysis required in classification development. A model includes, for example, the name and version of the classification, names of each category and their descriptions, possible translations, types of relations among categories, and mapping data regarding terminology harmonization [4,33,34].
2. Documentation of classification development: documenting the elements and progress of classification development in sufficient detail adds to the understanding of concept analysis and building relationships among categories, with an emphasis on clear separation among categories [4,16,33].
3. Multidisciplinary expertise: for example, clinical, terminological, informatics, and technical expertise used in the analysis and development process ensures relevant and usable outcomes [4,12,16,26,27].
4. Validation of results: development result validation based on study design and expertise or, for example, the translation of concepts and categories to ensure that the chosen concepts remain unchanged and the clinical meaning prevails. This supports interoperability and is especially relevant for small languages, such as Finnish [12,20,35,36].
5. Classification maintenance: after developing a classification, the formal governance model or informal maintenance process should include feedback from the clinicians (ie, the users) [34,37,38].

Classification development research is considered more practical than theoretically oriented, which results in development outcomes that might not necessarily be scalable to other research or use contexts [16,39,40]. Unsystematic and partial descriptions of classification development steps render it difficult to understand the underlying theoretical grounds for created structures and characteristics [33]. Applying a qualitative approach allows us to use somewhat limited research literature both as an analytical tool and to provide a source of concepts, theories, and hypotheses [20,31]. For qualitative research, a systematic review of literature is not typically required, although familiarity with relevant literature may increase sensitivity to subtle nuances of data [20,32]. In line with the qualitative approach [20], we identified key elements of classification development from the previous literature. The main literature consists of 12 research papers, and the results are summarized in Textbox 1. With this use case, we applied the elements found in the literature by researchers who described such elements and stages of classification development. Therefore, before embarking on classification development, typical elements based on the previous literature were compiled as guiding principles in the research process. These guiding principles were used and tested in practice during our recent study [12] (Textbox 1). However, these elements were not described in-depth in our publication.

According to the available literature, the classification development process begins with a careful, in-depth concept analysis to support the need for structured and controlled data representation (cf the study by Watson et al [16]). This is for documentation purposes [27] and for increasing the usability of data by ensuring improved data quality and comparability [41]. Reconciling clinical needs for documentation requires content analysis and mapping, which can be costly in both financial and temporal respects [4]. An important observation for classification development is that incomplete or overlapping conceptualizing, naming, and descriptions of categories and their relations within a classification may challenge resulting data quality owing to heterogeneity and indistinctness of concepts and terms used [4,33]. We have defined the concepts used in our research in the previous paper [12]. Our core concept regarding the phenomenon of the use case is a technology-induced error that results from the design and development of technology (Multimedia Appendix 2), the implementation and customization of technology, and the interplay between the operation of a technology and the new work processes that arise from the use of technology [42,43]. Regarding the classification category and subcategory building, our process was both qualitative and iterative [20]. The category building consisted of systematic analysis and labeling of varied phenomena illustrated in our data [20]. By examining differences and similarities in our data, we divided the data first into categories, iteratively refined distinctive differentiation between categories and within a category, and continued to define subcategories with an in-depth analysis.

From a methodological perspective, regarding the aim of improving systematic classification development, the potential strengths and weaknesses encountered during the process should be documented in research. Cornet et al [4] stated that content shortcomings encountered during classification development, for example, concept coverage and gaps, can be solved relatively easily. However, some formalism issues are more difficult to resolve. These include, for example, how relations among the categories are arranged, the overall structure or concept model, and how the classification is applied by the clinical users [27,41]. The United Nations suggests the following similar requirements in their practical guideline for classification development, for example, a consistent conceptual basis, a flat or hierarchic structure, categories that are mutually exclusive and exhaustive, and definitions that are clear and unambiguous and define the content of each category [44]. Furthermore, classifications should be relevant to users and sufficiently robust to last for a period of intended use. They should also provide comparability over time and among collections and provide guidelines for the coding and output of any data collected [36,38].

To summarize, as described in our study design, these elements form the basis for testing them in our use case.

## Results

### Overview

Our results provide examples of selected categories of our use case analysis to illustrate the development of technology-induced problem classification. During the analysis [12], we reviewed all patient safety incidents (N=1486) to identify technology-induced ones and continued to categorize the remaining incidents (n=501) with the concepts based on previous research. We added both main categories and subcategories to develop the initial content toward the context of mature EHRs according to evidence from the patient safety incident data. This required close cooperation between clinical and informatics experts within the research team to ensure that the categories captured identified phenomena correctly and were relevant for clinicians.

We provide the following four examples from our use case [12]: a description of the reasoning behind 2 categories, which were expanded to increase the potential accuracy of patient safety incident reporting, and 2 new categories, which were added to reduce ambiguities and any duplication of the original categories.

### Category of Usability Issues

The first example is illustrated by the category of usability issues (73/501, 17.1%). To start with, this category had no description, and the initial subcategories included features that were more closely related to other categories, such as documentation problems (60/501, 14.1%; Multimedia Appendix 2). With the help of clinical experts, we defined usability problems as situations in which the used EHR is difficult to use. For example, this complicates finding the required patient data or does not guide the clinician as expected, meaning that the system does not support the clinician’s work processes as expected according to care guidelines. In contrast, the documentation problems were defined to include a lack of data structure, errors, and ambiguities when entering data. After the analysis, we added 4 subcategories to usability issues, resulting in a 2-level hierarchy. Two of these subcategories (problems with decision support, n=2, and printing problems, n=11) were easily defined. For the other two subcategories (problems and deficiencies related to alarms, n=29, and problems with finding data, n=30), we added additional third-level subcategories. In addition, we added missing, incorrect, or difficult-to-interpret alarm and alarm fatigue to the first subcategory. For the second subcategory, we added that the information is difficult (illogical) for the user to perceive and the information is laborious to find and has to be dug up. These third-level subcategories were easily identified by the clinicians in our research team and are documented in previous research. Although they concern separate usability issues, only future use of the classification would reveal if the third-level subcategories are suitable for reporting. In an ideal situation, third-level subcategories could guide the reporting clinician to identify a usability issue more easily, based on the category-specific name and description. However, a deeper hierarchy of categories and additional subcategories could increase the reporting burden in fast-paced clinical work. To determine the most suitable level of accuracy for reporting, the pilot use of the developed classification would be required.

### Category of EHR Downtime

Regarding the category of EHR downtime (8/501, 1.9%), the starting point for classification development was a flat hierarchy, that is, a single-level main category, after which our development progressed iteratively. This category was identified as a relatively well-defined entity with no connection to other categories in the classification. Furthermore, it was known from the literature that domain coverage (including only planned and unplanned downtimes based on previous research at this stage) was insufficient to provide classification benefits. However, we sought a relatively simple 3-level hierarchical structure to facilitate clinical use. In addition to the literature, available real-world data in the form of patient safety incident reports guided more precise identification of new subcategories, including the completely new phenomenon of *the problem of logging on to a single application partly or entirely* (n=5). At this point, we divided the subcategory of the system logging problem in relation to the whole or part of the system in use to capture issues with a high-maturity EHR more effectively. The harmonization of concepts for known phenomena based on previous literature was relatively straightforward. However, the concepts for optimizing the new range of categories for clinical users required more reconciliation. Our aim is that categories would be mutually exclusive and exhaustive. During classification work with our data, we noticed that not all incidents fitted with existing subcategories. At this point, attention was given to content according to the feedback of clinicians as users. Without incorporating multidisciplinary knowledge, it would have been difficult to create and define content clearly and comprehensively, especially for the category *data entry during and after a downtime*.

### Category of Clinical Workflow Problems

During classification development, we added clinical workflow problems (33/501, 7.7%) as a new main category for two main reasons: the original classification addresses this phenomenon ambiguously, and mature EHRs implement clinical procedures according to defined workflow descriptions and guidelines. In other words, although building a workflow into EHRs is a well-established practice, the original classification left the issue either unidentified or partially identified. On the basis of the use case and the views of clinical experts, workflow problems can disrupt work procedure continuity. For example, although fundamental process steps such as a patient transfer or discharge should be carried out, the EHR renders it impossible to complete the procedure or does not otherwise support the clinician’s work as expected according to hospital guidelines. Moreover, owing to the lack of an integrated, functional, and logical workflow, the system may attract ways of clinical use that are not aligned with the guidelines when the expected EHR functionalities cannot be obtained. This can result in deviant work processes and workarounds. On the basis of our case data, the phenomena of workflow problems could be accurately identified and tentatively described. However, this could still benefit from insights from other use cases and data to confirm the correctness of our interpretation based on these specific data. Moreover, identifying workflow problems as a new main category can reduce the corruption of reported data caused by the weak distinctiveness of the original classification and its categories.

### Category of Medication Section Problems

We noticed that the original classification did not cover incidents related to the medication management section adequately, especially given that many of these cases were documented in the research data. Thus, a new main class of medication section problems (89/501, 20.8%) was added. These problems can result in situations where prescription and patient record information is not stored as intended, or where apparent changes occur because of an unidentified system-related reason. This is often a problem that hinders the management of overall medication. With this particular category based on its appearance in our research data, we wish to illustrate that there are some preconditions for classification development. First, the problems in the medication section appeared more difficult to delimit and describe compared with workflow problems. However, we recognized that these problems are closely related to the clinical workflow category features (eg, functionality of closed-loop medicine administration) and documentation category. Regarding medication section incidents, our research team concluded that there were still complex unresolved issues in the implementation of the new EHR from the implementation perspective, where immediate modification and mitigation appears to be necessary. Thus, it is likely that some of the system-specific problems will be solved. Moreover, when identifying real root causes to develop new subcategories, a more detailed examination would have been required, for example, through root cause analysis. In this case, it was too early to develop subcategories based on the research data; hence, the problem type could be identified but not unambiguously described. For this category, we found that a major functional change period, that is, system implementation in progress, may be a suboptimal time for developing a classification.

To conclude the insights from the use case, several iterations of category names and descriptions were required to ensure shared and sufficient understanding of the reasoning behind a specific category. Further, we used research literature to determine how the same or similar categories had been conceptualized. During classification development, clinician insights provided clinical understanding of a phenomenon through a clinical lens. However, informatics and IT expertise were required to identify and analyze system-specific starting points and boundaries of a specific phenomenon. Overall, our classification development was conducted through a sociotechnical lens to ensure comparable reasoning of both human and nonhuman factors that attribute to a specific technology-induced patient safety incident. However, when new categories or subcategories were constructed, the clinical perspective of relevant and usable classification content development remained a prevailing guideline. This was particularly true regarding clinical reporting and the optimal versus redundant level of accuracy for reporting based on the available categories and time available for reporting in hectic clinical work. To summarize, essential key elements, such as content analysis, category building, the definition of hierarchy among categories, and classification validation, provide requirements for systematic classification development.

## Discussion

### Principal Findings

On the basis of the study design, a set of guiding principles and preliminary elements for classification development were identified and applied in our use case. Next, we gathered evidence of the process for classification development and captured common elements based on previous research and our own experience of classification development. Finally, we processed these elements and explored examples from our recent research [12] in the *Results* section to illustrate the details of both the classifying process and elements to build sustainable and well-defined classifications in the future.

Our classification development and validation research [12] was used as a use case in this research to strengthen the fragmented methodological support for this type of research. In practice, we revealed incident-related gaps of conceptualization within the area of research. During classification development, filling these gaps meant analyzing various technology-induced patient safety incidents within our multidisciplinary research team. The incidents were analyzed with a sociotechnical lens to avoid bias when building the categories. Clinician insights into the incidents and the rationality of the clinical working environment played significant roles when identifying the categories.

Owing to the scarcity of research evidence, challenges when conducting this kind of research are recognized [4,12]. Accordingly, our main purpose is to fill a methodological gap in classification development research to promote well-grounded classification development. Although we ground these recommendations on the use case, the available research literature indicates similar challenges in other classification development cases. First, a concept model informing the classification development promotes systematic category building with structural formalism. In a way, such a model provides fundamental requirements for the development of each category. Second, in addition to conceptualizing category building, documenting agreements on other main elements can constitute guiding principles for the whole classification development. Furthermore, documenting common agreements can also support quality control and ensure the understanding of common goals for the development. Third, ensuring multidisciplinary expertise is crucial for guaranteeing that the intended clinical meaning prevails and for facilitating the validation of emerging classes. Moreover, regarding the future use of a classification under development, multidisciplinary expertise can be used to define the required level of category robustness and to review sufficient levels of clinical domain coverage and comprehensiveness. By reviewing clinical relevance and documenting practices during classification development, the development outcome might be feasible for clinical use. Fourth, terminological harmonization or systematic translation of concepts and terms representing the categories can be used to ensure that the clinical meaning remains unchanged during the development and that the chosen concepts remain relevant in the specific domain or clinical context. Fifth, planning ahead is a requirement of classification maintenance, especially when the clinical context in which the classification is used is constantly changing and evolving. In this regard, planning for user feedback or other routes for receiving and processing development needs emerging from the clinical context and users in the future can provide a means of classification maintenance.

### Limitations

Any method of qualitative or quantitative analysis is not a purely technical process, as it is inevitably influenced by the scientific background and experience of researchers. Accordingly, critical reflection throughout the research process is of considerable importance. The multidisciplinary research team with prior training and experience in qualitative analysis worked closely to follow up on research progress analytically and critically to ensure the quality of the conduct of the study. However, there are many known weaknesses associated with the qualitative research approach in particular [20,30-32]. In this study, the approach was iterative, the analysis was interpretative, and the results were consequently descriptive, which is typical of this research approach. Therefore, the results should be considered from the outset of these facts. The most difficult methodological question arose from the situation where the researchers determined that a systematic review of the literature in this area does not necessarily produce such results that would benefit the achievement of the research goals. Moreover, the researchers have been working on governmental and international classification development for many years, which contributed to the decision to proceed in a way that deviated from the original research plan, which relied on the possibilities provided by the qualitative research literature. Furthermore, the literature was studied and adopted cumulatively.

The theoretical framework for our classification development was the sociotechnical model [9,10]. It should be noted that classification development can be based on many starting points and can occur within many themes. As a result, we cannot conclude from this use case alone that our approach would be applicable to all subject areas. This is why further research is still required, in which our observations could be tested with new data.

### Lessons Learned and Implications for Future Work

There are many possible aspects for future classification development work and related research based on our study results. Based on our observations, for the continuum of classification development in each context, we suggest that sufficient multi-professional analysis and review should be part of classification development and maintenance. Overall, the classification development should place more emphasis on terminological and clinical subject area expertise.

Moreover, classifications can be regarded as a representation framework among evolving practices, meaning they must be followed by new knowledge. Furthermore, more case or pilot studies with real-world contexts are required. Our use case and developed classification serve as an example here: patient safety incident data retrieved from other source EHRs and use contexts could strengthen the methodological approach or it might contribute to further development of our classification based on user feedback.

As we became acquainted with the classification literature, preliminary observations were made about promising future possibilities, specifically of ontologies. Although we excluded the topic from our own research, we highlight the potential for future research. Hancock [41] described a study that demonstrates the demand for instant data and information is enabled through innovative and newer ways of classifying information. From a system perspective, much of the development process can now be automated, content can be contributed and approved on the web, and computer programs are sufficiently advanced to consider more human-thinking methodologies. Although it will take time to replace traditional approaches to classification development and theory with innovative, technological solutions, we suggest re-examining the classification development process we have described, starting with the concept definition phase. It would be particularly interesting to determine whether machine learning can be used in this context, especially if the aim is to analyze extensive data sets.

### Conclusions

Although classifications remain significant tools for clinical documentation and for producing clinical data in various clinical domains, limited research literature is available that illustrates classification development systematically or from a methodological perspective based on previous research. Owing to the role of classifications in data production, theoretical and systematic reviews could also contribute to the transparent development of a future health care knowledge base. Thus, we recommend the main elements based on this study for systematic classification development. Furthermore, the research presented herein could serve as a basis for future work. To conclude, there is a need for the scientific assessment of whether classifications in different domain areas can be developed from theoretical and systematic perspectives.

## Appendix

Multimedia Appendix 1 Summary of the study design.

Multimedia Appendix 2 Concept model of the use case specific classification development.

## Abbreviations

EHR: electronic health record
IT: information technology
