# Supplementary material for: Preliminary Exploration of Main Elements for Systematic Classification Development: Case Study of Patient Safety Incidents
Source: JMIR Form Res. 2022 Mar 29;6(3):e35474. doi: 10.2196/35474 (PMC9006139; doi:10.2196/35474)
Supplement: Multimedia Appendix 1 [file formative_v6i3e35474_app1.docx]

Multimedia appendix 1

Summary of study design
